# Swept-sine noise-induced damage as a hearing loss model for preclinical assays

**DOI:** 10.3389/fnagi.2015.00007

**Published:** 2015-02-16

**Authors:** Lorena Sanz, Silvia Murillo-Cuesta, Pedro Cobo, Rafael Cediel-Algovia, Julio Contreras, Teresa Rivera, Isabel Varela-Nieto, Carlos Avendaño

**Affiliations:** ^1^Institute for Biomedical Research “Alberto Sols” (IIBM), Spanish National Research Council–Autonomous University of Madrid (CSIC-UAM)Madrid, Spain; ^2^Centre for Biomedical Network Research (CIBER), Institute of Health Carlos III (ISCIII)Madrid, Spain; ^3^Príncipe de Asturias University Hospital, University of Alcalá, Alcalá de HenaresMadrid, Spain; ^4^Hospital La Paz Institute for Health Research (IdiPAZ)Madrid, Spain; ^5^Institute for Physical and Information Technologies (ITEFI), Spanish National Research Council (CSIC)Madrid, Spain; ^6^Veterinary Faculty, Complutense University of MadridMadrid, Spain; ^7^Department of Anatomy, Histology and Neuroscience, Medical School, Autónoma University of MadridMadrid, Spain

**Keywords:** cytocochleogram, hair cells, hearing loss, transtympanic, TGF-β inhibition, violet noise

## Abstract

Mouse models are key tools for studying cochlear alterations in noise-induced hearing loss (NIHL) and for evaluating new therapies. Stimuli used to induce deafness in mice are usually white and octave band noises that include very low frequencies, considering the large mouse auditory range. We designed different sound stimuli, enriched in frequencies up to 20 kHz (“violet” noises) to examine their impact on hearing thresholds and cochlear cytoarchitecture after short exposure. In addition, we developed a cytocochleogram to quantitatively assess the ensuing structural degeneration and its functional correlation. Finally, we used this mouse model and cochleogram procedure to evaluate the potential therapeutic effect of transforming growth factor β1 (TGF-β1) inhibitors P17 and P144 on NIHL. CBA mice were exposed to violet swept-sine noise (VS) with different frequency ranges (2–20 or 9–13 kHz) and levels (105 or 120 dB SPL) for 30 min. Mice were evaluated by auditory brainstem response (ABR) and otoacoustic emission tests prior to and 2, 14 and 28 days after noise exposure. Cochlear pathology was assessed with gross histology; hair cell number was estimated by a stereological counting method. Our results indicate that functional and morphological changes induced by VS depend on the sound level and frequency composition. Partial hearing recovery followed the exposure to 105 dB SPL, whereas permanent cochlear damage resulted from the exposure to 120 dB SPL. Exposure to 9–13 kHz noise caused an auditory threshold shift (TS) in those frequencies that correlated with hair cell loss in the corresponding areas of the cochlea that were spotted on the cytocochleogram. In summary, we present mouse models of NIHL, which depending on the sound properties of the noise, cause different degrees of cochlear damage, and could therefore be used to study molecules which are potential players in hearing loss protection and repair.

## Introduction

Noise-induced hearing loss (NIHL) is the most common form of acquired deafness in developed countries and therefore it is a public health priority (Sliwinska-Kowalska and Davis, 2012). Hearing impairment may be induced by a single impulsive noise or after repetitive exposure to moderate or high intensity noise (Kirchner et al., 2012). The cumulative damaging effects on the inner ear depend on the noise characteristics (frequency, level), chronicity and individual susceptibility to noise (Konings et al., 2009; Le Prell, 2012). Inner and particularly outer hair cells (IHC and OHC, respectively) especially those located in the basal turn of the cochlea in mammals, are very sensitive to noise damage. Thus, the disruption of stereocilia leads to a severe alteration in HC structural integrity that has been correlated to permanent threshold shifts (TS) in many species (Hamernik and Qiu, 2000; Chen and Fechter, 2003; Chen et al., 2003; Hu et al., 2006; Bohne et al., 2007; Harding and Bohne, 2009).

Mice models are essential tools for studying the pathophysiology of NIHL and for evaluating new potential therapies (Ohlemiller, 2006; Park et al., 2013). As reported in human cases, functional and structural alterations depend on noise level and duration of exposure, and also on strain susceptibility. Thus, C57BL/6 and BALB/c mice are particularly vulnerable to noise, whereas CBA/Ca mice appear to be more resistant (Ohlemiller et al., 2000, 2011; Ou et al., 2000a; Gratton et al., 2011). There are many reports on the functional and morphological characterization of different NIHL mouse models in which mice are usually exposed for a few (1–4) hours to high intensity (95–120 dB SPL) white or octave band noises, usually centered on low frequencies up to 10 kHz (Park et al., 2013). These frequencies are in the lowest frequency range of the mouse hearing spectrum (Greenwood, 1996). Exposures to noise over 10 kHz are much less frequent (i.e., 8–16 kHz in Kujawa and Liberman, 2009; 4–45 kHz in Ohlemiller et al., 2011). In this work we generated NIHL by short exposure to “violet” swept-sine noise (VS), a 10 s linear sweep in frequencies from 2 kHz to 20 kHz designed with high-pass filtering and linear with frequency gain (Cobo et al., 2009). Additionally VS noise with frequencies from just 9 to 13 kHz was used to injure a specific stretch of the basilar membrane (BM). To illustrate and quantify the distribution of HC along the length of the cochlea under the different conditions used, a cytocochleogram was performed, applying a stereological approach to previously published procedures (Ou et al., 2000b; Viberg and Canlon, 2004; Müller and Smolders, 2005; Müller et al., 2005; Boyce et al., 2010).

Here we describe the functional and morphological consequences, and their correlation, of exposure to VS noises of different sound levels and frequency ranges. We found that the characteristics of the VS noise determine the pattern of hair cell density along the cochlea and, accordingly, the ABR electrophysiological response. Therefore, the study of NIHL and of potential prevention and repair therapies could be further improved by refining the design of noise stimuli in experimental models. Furthermore, we tested the local actions of two peptides P17 or P144 inhibitors of transforming growth factor beta 1 (TGF-β1) to repair NIHL. TGF-β1 is a cytokine involved in the cochlear inflammatory response that is released early after cochlear injury caused by aminoglycoside (Wissel et al., 2006), antigens (Satoh et al., 2006), otitis media (Ghaheri et al., 2007) and noise exposure (Murillo-Cuesta et al., submitted). Our functional and cochleogram data indicated that a single local administration of either peptide did not exert a therapeutic effect on NIHL.

## Materials and methods

### Mouse housing and handling

The study was carried out on 2 month old male mice from CBA/CaOlaHsd (CBA) and C57BL/6JOlaHsd (C57) strains (Harlan Laboratories). Patterns of cochlear damage after noise exposure in these strains have been previously described; both strains show consistent lesions but different susceptibilities to noise injury (Ohlemiller et al., 2000, 2011; Ou et al., 2000a; Wang et al., 2002; Ohlemiller and Gagnon, 2007; Park et al., 2013). All procedures and animal handling were conducted in accordance with the European (2010/63/EU) and Spanish (RD 1201/2005) legislation on the experimental use of animals, and were approved by the Ethics Committee of the Spain National Research Council (CSIC).

### Hearing evaluation

**Figure 1:**
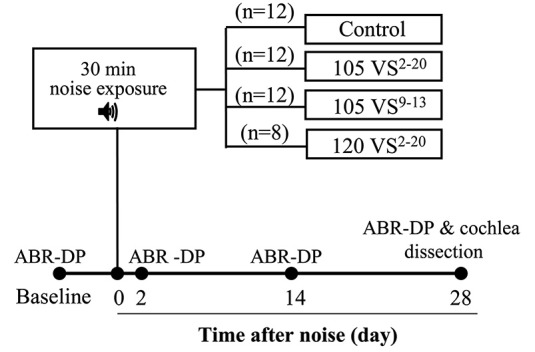
**Chronogram of the experiment**. Mice were exposed to Violet Swept-sine (VS) noise with different intensities (105 or 120 dB SPL) and frequency ranges (2–20 or 9–13 kHz) for 30 min. Hearing was evaluated with Auditory Brainstem Responses (ABR) and Distortion Products of Otoacoustic Emissions (DP) before (baseline) and 2, 14 and 28 days after noise exposure. At the end of the study, cochleae were dissected and processed for histology and cell counting.

Auditory brainstem responses (ABR) and distortion product otoacoustic emissions (DPOAE) were obtained before and 2, 14 days and 28 days after noise exposure (Figure 1) with a System III Evoked Potential Workstation (Tucker-Davis Technologies, Alachua, FL, USA). Briefly, mice were anesthetized with ketamine (Imalgene, Merial, 100 mg/kg) and xilacyne (Rompun, Bayer, 10 mg/kg) and placed on a heating pad inside a soundproof acoustic chamber.

For the ABR test, click and tone burst stimuli (8, 16, 20, 28 and 40 kHz) were presented with an MF1 magnetic speaker (TDT) from 90 to 20 dB SPL in 5–10 dB SPL steps (Cediel et al., 2006; Riquelme et al., 2010; Murillo-Cuesta et al., 2012). Click stimuli were 0.1 ms and tone burst stimuli were 5 ms duration (2.5 ms each for rise and decay, without plateau). Threshold of click-evoked and tone-evoked ABR, peak latencies and amplitudes were determined. For DPOAE, an ER10B+ probe (Etymotic Research Inc., IL, USA) was inserted into the external auditory canal and mice were stimulated with two synchronic tones, whose frequencies (f1, f2; relation f1/f2 = 1.2) were calculated from a central frequency (*F* = 8, 10, 14, 18 and 22 kHz; f1 = F*0.909, f2 = F*1.09), and presented with decreasing intensity from 80 to 30 dB SPL (f1 level = f2 level). The distortion product 2f1–f2 was determined for each sound level from the FFT waveforms. DPOAE thresholds were defined as the minimum level of the primary tones that elicit a 2f1–f2 response higher than background noise.

### Noise exposure and experimental groups

Mice were exposed for 30 min to noise in a reverberant chamber acoustically designed to reach maximum sound level with minimum deviation in the central exposure area (Cobo et al., 2009). Two sound stimuli, named violet (V) and violet swept-sine (VS), were designed with Wavelab Lite software (Steinberg Media Technologies GmbH, Hamburg, Germany). Both have a linear-with-frequency gain to compensate for the high frequency losses inside the chamber, and present a spectrum biased towards high frequencies, a kind of violet coloring. V noise was synthesized from white noise, whereas VS noise was a 10 s linear sweep in frequency from 2 kHz up to 20 kHz that was repeated during the 30 min of exposure. Specific characteristics of these stimuli are described in Cobo et al. (2009).

Preliminary experiments with V and VS noise were conducted in C57 and CBA mice in order to select the appropriate mouse strain and noise. For further studies, CBA mice were used and four experimental groups were established (Figure 1). Mice of the control group were not exposed (*n* = 12) whereas other mice were exposed to VS noise with the following level and frequency ranges: 105 dB SPL and 2–20 kHz (105 VS^2–20^, *n* = 12), 120 dB SPL and 2–20 kHz (120 VS^2–20^, *n* = 8), and 105 dB SPL and 9–13 kHz (105 VS^9–13^, *n* = 12).

Finally, to evaluate the efficacy of P17 and P144 peptides, mice were exposed to 105 VS^2–20^ for 30 min and operated on 48 h after noise damage, once the increase in hearing thresholds was confirmed. The effect of noise exposure on hearing function was evaluated with the ABR test as described before, 2, 14 and 28 days after noise exposure.

### Drug administration

Chemically synthesized peptide inhibitors with high affinity for TGF-β1 (Ezquerro et al., 2003) were gently defrosted, diluted and sonicated (only P144) to completely dissolve them. Local administration of inhibitors into the inner ear was performed 24 h after noise exposure, once the increase in hearing thresholds was confirmed. Briefly, the tympanic bulla was exposed via ventral surgical approach, and a bullostomy was performed at the posterolateral aspect using a small hook. Once the round window and stapedial artery were clearly visible, we applied directly 10 µl of a concentrated (40 mg/ml) P17 or P144 solution or saline (*n* = 6 each) to the round window using a gelatin sponge vehicle (Murillo-Cuesta et al., 2009).

### Cochlear processing for histology and hair cell counting

**Figure 2:**
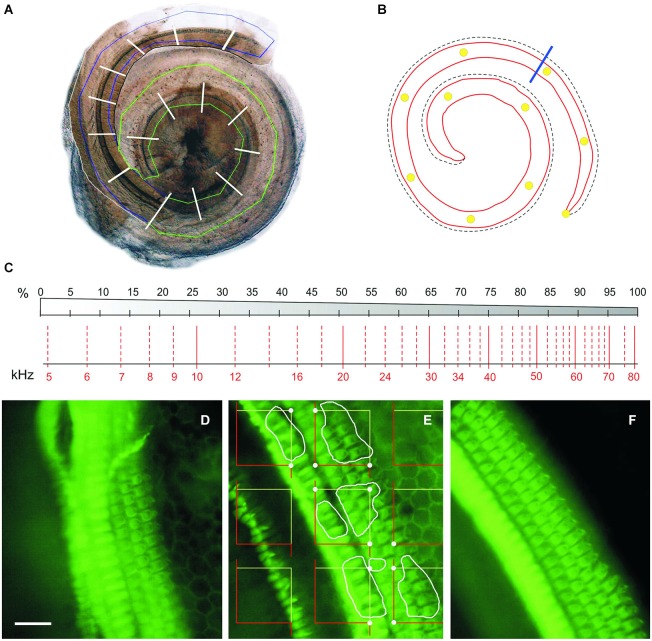
**Cochleogram and stereology. (A)** Photomontage of digital images of the two blocks of a CBA mouse dissected cochlea. The region containing the BM is roughly delineated in green (apical-middle block) or blue (basal block); white traverses divide the whole length of the cochlea into 5% intervals. **(B)** Diagram of the BM of the same cochlea, with 10% intervals marked with yellow dots. The blue line separates the portion reconstructed in A (roughly 80% from the apex) from the basal quarter-turn, which was never included in the reconstruction. **(C)** Diagram showing a place-frequency map according to Viberg and Canlon (2004) and Müller et al. (2005), which combines a schematic drawing of the BM divided in 5% bins (top) and the corresponding frequency map (bottom). **(D–F)** Phalloidin-stained whole mounts of portions of the apical **(D)**, middle **(E)** and basal **(F)** cochlear turns in a control mouse. The stereological method used to estimate HC densities is summarized in **(E)**: A set of unbiased frames is randomly placed on the *stereociliary fringe*, the convex hull area bounding the hair tufts of the IHC and OHC; the HCs to be included in the count in this example are delineated by white profiles; dots highlight the frame corners that hit the stereociliary fringe, and serve to estimate the reference space sampled with the frames. Scale: 25 µm. IHC, inner hair cells; OHC, outer hair cells.

At the end of the experiment (Figure 1), mice were anesthetized with pentobarbital (Dolethal, Bayer, 150 mg/kg) and cochleae were extracted for light microscopy or HC counting. For histological evaluation, mice were perfused with 4% paraformaldehyde (in 0.1 M saline buffer, pH 7.4) and the cochleae were removed, fixed overnight, decalcified with 10% EDTA (0.3 M, pH 6.5) and embedded in paraffin as described (Riquelme et al., 2010; Murillo-Cuesta et al., 2012). Mid-modiolar 10 µm sections were Nissl-stained and evaluated with a Zeiss Diaplan microscope and a digital camera (Leitz DFC300 FXC). For HC counting, mice were sacrificed by cervical dislocation and the inner ear was carefully dissected. After removing the bony wall of the tympanic bulla and the stapes, the cochlea was exposed and two openings were performed, one between the round and oval windows and one in the apex, to circulate 300 µl paraformaldehyde. The cochleae were immersed in paraformaldehyde for 24 h and decalcified with EDTA for 4–6 days. Using an angled sharp micro scalpel, the bony and membranous labyrinths and the tectorial membrane were carefully removed to expose the organ of Corti (OC). Total removal of the BM along the entire cochlea is technically difficult; the basal-most region or “hook” is more delicate and it is easily injured during dissection. Therefore, the cochleogram shown in this work represents the 80% (range across cases: 70–85%) of the cochlea that can be dissected while maintaining cellular integrity. The 20% of the cochlea which is destroyed results in a loss of information regarding the 50–80 kHz frequencies, which, by definition, should not be greatly affected by noise containing frequencies in the range of 2–20 kHz (Figure 2).

OC was sectioned into two portions, one containing the apex and middle turns and the other containing the basal turn of the cochlea, placed on plates (Nunclon Microwell Plates, Sigma Aldrich) filled with PBS, and stored at 4°C. Samples were permeabilized with 0.5% Triton X-100 (Sigma-Aldrich) for 15 min, at room temperature on a shaker, and then incubated 1 h with 1:1000 phalloidin (R Alexa Fluor 488 phalloidin, Invitrogen) in PBS. After three washes in PBS, the samples were transferred to concave microscope slides (Menzel-Gläser) and mounted with Vectashield mounting medium with DAPI (Vector Laboratories). A total of 43 cochleae were obtained from the 22 animals (105 VS^2–20^, *n* = 6), 120 VS^2–20^, *n* = 6, 105 VS^9–13^, *n* = 6, control non-exposed mice, *n* = 4).

### Stereology and cochleogram plotting

In order to plot a standardized cochleogram and to correlate cell damage with increased hearing thresholds at specific frequencies, a stereological approach was designed to determine the number and distribution of HCs along the length of the cochlea (Viberg and Canlon, 2004; Müller et al., 2005; Boyce et al., 2010; Figure 2). “Flattening” of the BM that resulted from cochlear extraction and mounting procedure helped to ensure an unbiased sampling of cells. High resolution photomicrographs of the OC samples were taken through a BX51 microscope with a DP70 camera (Olympus). These images were studied offline using CorelDRAW X3 software (Corel Corp., Ottawa, Ontario, Canada) to define an area of interest along the BM that included the rows of HCs, This area was further divided into sectors, each covering 5% of the total length, from apex to basal region. The OC samples were then evaluated with the same microscope under ultraviolet epi-illumination (Olympus U-RFL-T) with 4X and 40X magnification to detect phalloidin immunofluorescence, and previously defined sectors were located in the sample in order to perform cell counts. Interactive test grids and control of the motorized stage (Prior Scientific, Rockland, MA, USA) were provided by CAST stereological software package (v.2.3.2.0, Visiopharm, Hørsholm, Denmark). HC loss and stereocilia abnormalities are thought of as the principal correlate in NIHL. Since each HC is unequivocally represented by its hair bundle, phalloidin-stained stereocilia were used as counting units and HC were counted as present if the hair bundle was intact.

HC count was carried out by placing unbiased frames in a uniformly random sampling strategy on the focus planes of the cilia. The reference space was considered the *stereociliary fringe*, that is, the convex hull area bounding the region containing the rows of hair tufts of the HCs.

The IHC and OHC cell densities were estimated for each sector as follows,

$$N_{A}\left(HC\right)=\frac{\sum Q\left(HC\right)\cdot 1000}{\sum a\left(SF\right)}$$

where *N_A_* is the cell density expressed as number of cells/1000 µm^2^, *∑Q* is the sum of HC counted within each sector, and *∑a(SF)* is the area of interest, in µm^2^, sampled with the unbiased frames within the given sector. The precision of this method was approximated by computing the coefficient of error (CE) of the estimates (N_A_) obtained on each sector, applying Cochran’s equation for ratio estimators (eq. 10.32 in Howard and Reed, 2005). Our strategy yielded CE values of ~17% (0.17 ± 0.015) in the control animals, and ~21% (0.21 ± 0.020) in the noise-damaged groups.

Cytocochleograms were constructed by plotting the number of present OHCs or IHCs, or the OHC and IHC densities, as a function of percent distance from the apex of the cochlea.

### Statistical analysis

Data obtained from hearing test and hair cell counting were analyzed using v.19 IBM SPSS Statistics software. Results were expressed as mean ± SEM. Differences in ABR and DPOAE thresholds were analyzed using analysis of variance (ANOVA) with repeated measures. Bonferroni and T2 Tamhane *post hoc* tests (for equal or unequal variances, respectively) were used to make pairwise comparisons of the repeatedly measured data in different measurement times for each experimental group. ANOVA tests were also performed for comparison of HC numbers and densities among experimental groups. Results were considered significant at *p* ≤ 0.05.

## Results

### Longitudinal study of hearing-loss after exposure to different noise types

Preliminary experiments were conducted in C57 and CBA mice in order to select the appropriate noise for subsequent cytocochleogram studies. Mice were exposed to noise for 30 min and threshold of click-evoked and tone-evoked ABR were determined before and at 1 h, and 3 and 7 days after exposure to noise. Results are summarized in Table 1. C57 mice exposed to noise at 120 dB SPL showed severe cochlear damage with irreversible TS, whereas following exposure at 100 dB SPL, baseline thresholds were practically recovered in the first week after insult, especially when V noise was used. CBA mice exposed to VS 105 dB SPL noise showed smaller TS than C57 mice, thus CBA was the strain chosen for further studies.

**Table 1:** **Evolution of ABR threshold in response to click stimulus**.

				ABR threshold (dB SPL)			
					Time after noise exposure		
Strain	Stimulus	Level (dB SPL)	N	Baseline	1 h	3 days	7 days
C57BL/6JOlaHsd	V^2–20^	100	5	42 ± 3	55 ± 10	48 ± 12	41 ± 2
		120	3	43 ± 8	90 ± 10	90 ± 17	87 ± 12
	VS^2–20^	100	4	42 ± 6	63 ± 5	60 ± 8	53 ± 5
		120	4	40 ± 8	88 ± 10	90 ± 8	83 ± 17
CBA/CaOlaHsd	VS^2–20^	105	12	17 ± 5	n.d.	69 ± 4	70 ± 6

*Exposure of mice to noxious noises at 100 dB SPL for 30 min induced moderate TS with almost complete recovery, especially when V^2–20^ noise was used. In contrast, 120 dB SPL noise exposure irreversibly damaged the cochlea. Exposure to VS^2–20^ noise in CBA mice was further selected to study functional effects and concomitant hair cell loss. V, violet noise; VS, violet swept-sine noise. Superscripts indicate the noise frequency range (in kHz)*.

**Figure 3:**
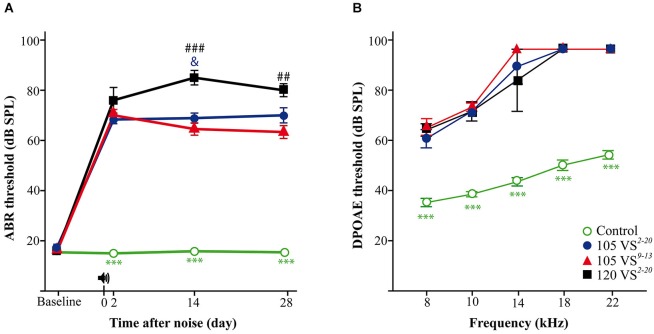
**Evaluation of hearing. (A)** Evolution of threshold of click-evoked ABR. Control non-exposed mice (*n* = 12) maintained normal hearing thresholds along the study, with statistically significant differences (*p* < 0.001, ***) compared to noise damaged mice. Two days after noise insult, a notable TS occurred in exposed animals; at longer post-exposure periods, mice exposed to 120 VS^2–20^ (*n* = 7) showed significantly higher values compared to those exposed to 105 VS^2–20^ (*n* = 11, *p* < 0.05, &) and 105 VS^9–13^ (*n* = 12, *p* < 0.01, ##). **(B)** DPOAE thresholds at individual frequencies at the end of the experiment. 28 days after noise exposure (105 VS^2–20^, *n* = 5; 105 VS^9–13^, *n* = 8; 120 VS^2–20^, *n* = 4) mice showed significant (*p* < 0.001, ***) higher DPOAE thresholds for all the frequencies as compared to control mice (*n* = 6). No significant differences were found among exposed animals. VS, violet swept-sine noise; superscripts indicate the noise frequency range (in kHz); the coefficient indicates the noise level in dB SPL.

In these advanced studies, CBA mice from the four experimental groups (Figure 1) showed baseline thresholds of click-evoked and tone-evoked ABR below 20 dB SPL, without significant differences among them (Figure 3A). The control group maintained normal hearing thresholds throughout the experiment, with highly significant differences (Wald $\chi_{\left(3\right)}^{2}$ = 2282.9, *p* = 0.000) when compared to all groups of noise-exposed mice. A click TS of around 50 dB occurred 2 days after noise exposure in all mice exposed to VS noise. No significant differences were found among damaged groups, although mice with the greatest TS were those exposed to 120 dB SPL noise. The evolution of click thresholds presented specific patterns for the three noise-exposed groups (Figure 3A). Thus, mice exposed to 120 VS^2–20^ noise showed a worsening of threshold 2 weeks after damage, with significant differences compared to exposure to 105 VS^2–20^ (*F*_(3)_ = 202.64, *p* = 0.026) and 105 VS^9–13^ (*p* = 0.000) noises. No differences were found between 105 VS^2–20^ and 105 VS^9–13^ noises at any time after insult, although the latter showed a non-significant but consistent small recovery of click thresholds. Similarly, baseline DPOAE thresholds were similar in all the experimental groups. Noise-exposed mice showed marked threshold increases in all the tested frequencies, with statistically significant differences compared to the control group, but without evident differences among them (Figure 3B).

**Figure 4:**
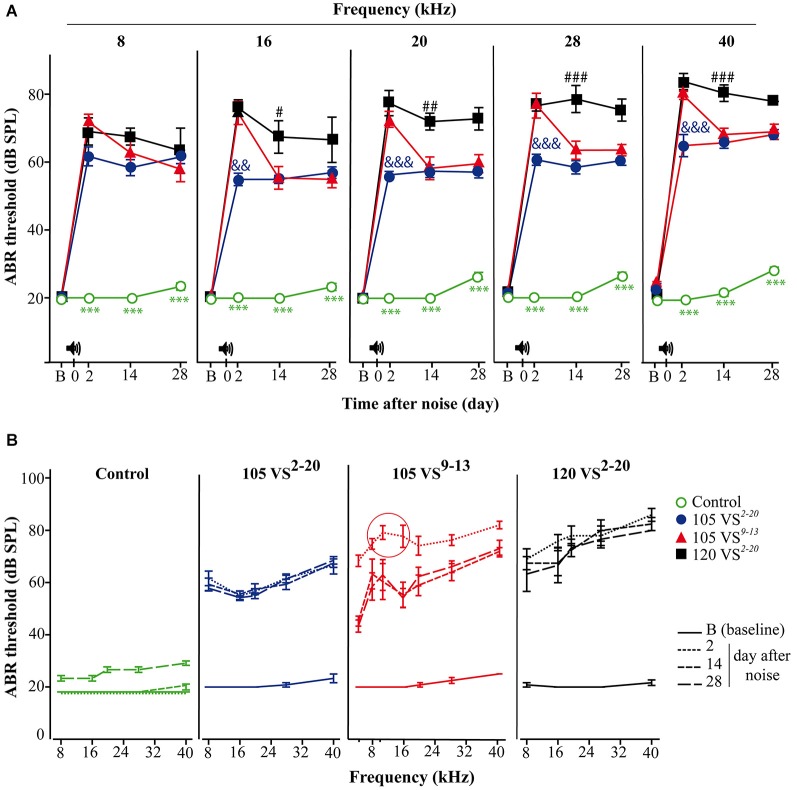
**Evolution of thresholds of tone-evoked ABR. (A)** Control non-exposed mice (○, *n* = 12) maintained baseline **(B)** hearing thresholds and showed statistically significant differences (*p* < 0.001, ***) when compared to noise-damaged mice. Noise 120 VS^2–20^ (■, *n* = 7) induced the most severe damage (compared to 105 VS noises; # *p* < 0.05, ## *p* < 0.01, ### *p* < 0.001), whereas TSs in 105 VS^2–20^ (●, *n* = 11,) were significantly lower (compared to the other noises; && *p* < 0.01, &&& *p* < 0.001). Mice exposed to 105 VS^9–13^ (▲, *n* = 12,) showed significant early damage followed by a partial, but quick, recovery. **(B)** Mice exposed to VS noise showed an elevation in the audiogram, compared to control mice. A TS peak in the 105 VS^9–13^ was evident in response to 8 and 10 kHz. VS, violet swept-sine noise; superscripts indicate the noise frequency range (in kHz); the coefficient indicates the noise level in dB SPL.

In contrast, differences were found in the audiogram between exposure to 105 VS^2–20^ and 105 VS^9–13^ noises. Thus, mice exposed to VS^9–13^ noise showed higher TS 2-days after noise exposure, especially when high frequencies were tested (Figure 4A). Significant differences were found at stimuli frequencies of 16 (*F*_(3)_ = 32.7, *p* = 0.002), 20 (*F*_(3)_ = 94.6, *p* = 0.000), 28 (*F*_(3)_ = 86.7, *p* = 0.000) and 40 (*F*_(3)_ = 177.1, *p* = 0.000) kHz.

To study potentially selective damage on specific cochlear regions, the audiogram of mice exposed to VS^9–13^ noise was extended to include frequencies of 4 and 10 kHz. As expected, the TS at 10 kHz was larger than TS found at the nearby frequencies of 4, 8 and 16 kHz. This TS peak in the audiogram was maintained throughout the study (Figure 4B).

Statistical analysis of ABR latencies showed a significant decrease of the peak and interpeak latencies, especially I–II, in animals exposed to noise compared to baseline values. This reduction was similar in the three exposed groups, without significant differences, and persisted throughout the times examined (data not shown).

### Cochlear morphology and hair cell density after noise exposure

**Figure 5:**
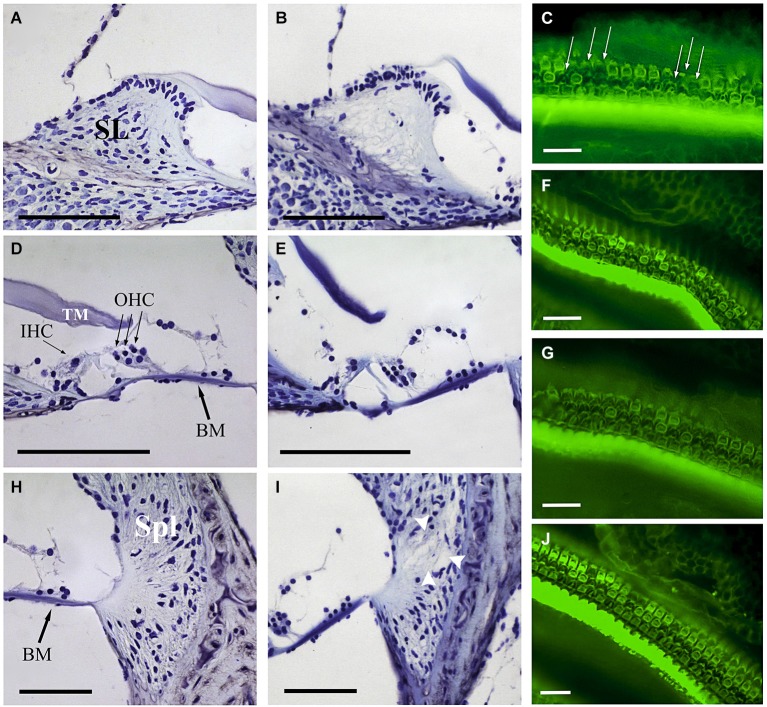
**Comparative study of cochlear morphology after noise insult**. Comparison of the spiral limbus (SL) **(A,B)**, organ of Corti **(D,E)** and spiral ligament (Spl) **(H,I)** of control **(A,D,H)** and VS noise-exposed **(B,E,I)** mice at the cochlear middle level. Main features observed after short time noise exposure included fibrocyte loss at SL **(B)** and loss of type IV fibrocytes at Spl (white arrowheads at **I**). The organ of Corti reveals an essentially normal gross anatomy (no collapse of tunnel of Corti, Hensen cells or spaces of Nuel). The different noise exposure conditions show similar cytoarchitectural alterations with no evident differences among them. At the focal plane shown, phalloidin-stained whole mounts for actin exhibit the disrupted sequence (arrows in **C**) of otherwise well-defined outlines of OHC and a substantial OHC loss 28 days after VS noise insult **(C,F,G,J)**. Scale bars: 100 µm **(A,B,D,E,H,I)**, 25 µm **(C,F,G,J)**. BM, basilar membrane; IHC, inner hair cells; OHC, outer hair cells; TM, tectorial membrane; SL, spiral limbus; Spl, spiral ligament.

**Figure 6:**
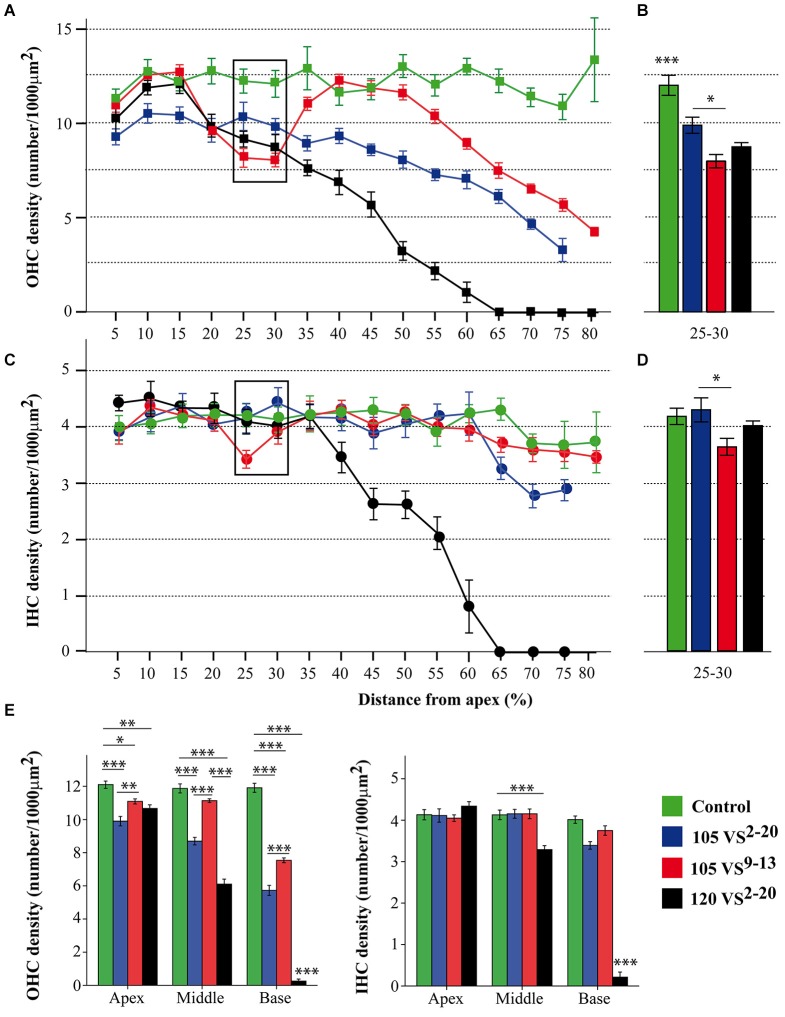
**Hair cell counting data. (A–D)** Inner and outer hair cell density (mean ± SEM, expressed as number of cells/1000 µm^2^) for each 5% sector across the total length of the BM **(A,C)** and focused on sectors 25–30 **(B,D)**. Exposure to 105 VS^9–13^ noise induced a significantly higher loss of hair cells in the cochlear region corresponding to sectors 25 and 30 (see Figure 2C) compared to exposure to 105 VS^2–20^ noise. **(E)** To better assess regional variations, sectors were grouped into three regions (apex, 5–25; middle, 30–55; base, 60–80) and the mean hair cell density was calculated. Statistically significant differences among groups were found, indicating that exposure to 120 VS^2–20^ noise induced the most severe damage, especially towards the basal region. * *p* < 0.05; ** *p* < 0.01; *** *p* < 0.001. VS, violet swept-sine noise; superscripts indicate the noise frequency range (in kHz); the coefficient indicates the noise level in dB SPL.

At the end of the experiment, CBA mice exposed for 30 min to VS stimulus showed some of the typical chronic cochlear alterations reported in this strain after noise damage (Wang et al., 2002); no gross differences were detected among 105 VS^2–20^, 120 VS^2–20^ and 105 VS^9–13^ groups. The main cellular feature detected in Nissl-stained sections was fibrocyte loss in the spiral ligament and spiral limbus (Figure 5), whereas no evident changes in the gross anatomy of the OC were observed. Stereocilia damage and HC loss were in fact evident in phalloidin-stained whole mount samples (Figures 5C,F,G,J). These preparations were further used for stereological hair cell counting. Figure 2 shows that the whole length of the dissected BM was divided into 5% sectors from apical to basilar cochlear regions. Present IHC and OHC were counted and densities were estimated for each sector in control and noise exposed mice (Figure 6). Control non-exposed mice showed similar hair cell density values in all sectors, both for OHC (over 10 cell/1000 µm^2^) and IHC (over 4 cell/1000 µm^2^). Differences among noise-damaged mice were evident from sector 20 onwards, especially for OHC. Mice exposed to 120 VS^2–20^ noise showed a clear decrease in OHC density, mainly at the basal region, where no OHCs were detected. A similar but smaller density decrease was observed in 105 VS^2–20^ exposed mice when compared to those exposed to 120 VS^2–20^. Whereas mice exposed to 105 VS^9–13^ noise showed an acute loss of OHCs in sectors 25 to 30%, and a progressive decrease of cellular density from sector 55% onwards (Figures 6A,B). Similar results were observed when IHC density was evaluated in all the experimental groups (Figures 6C,D).

Sectors were grouped into three cochlear regions (apical, 5–25%; middle, 30–55%; basal, 60–80%) for comparison (Figure 6E). As expected, the control group presented higher HC densities in the three regions compared to noise-exposed mice. A notable decrease in OHC density was observed in mice exposed to 120 VS noise, especially in the middle and basal regions. Mice exposed to 105 VS^2–20^ noise showed a gradual decrease of OHC density from apex to base, whereas in the group of mice exposed to 105 VS^9–13^ noise, OHC loss was evident in the basal region when compared to non-exposed mice (*p* = 0.000).

### Evaluation of TGF-β1 inhibitors in the treatment of NIHL

**Figure 7:**
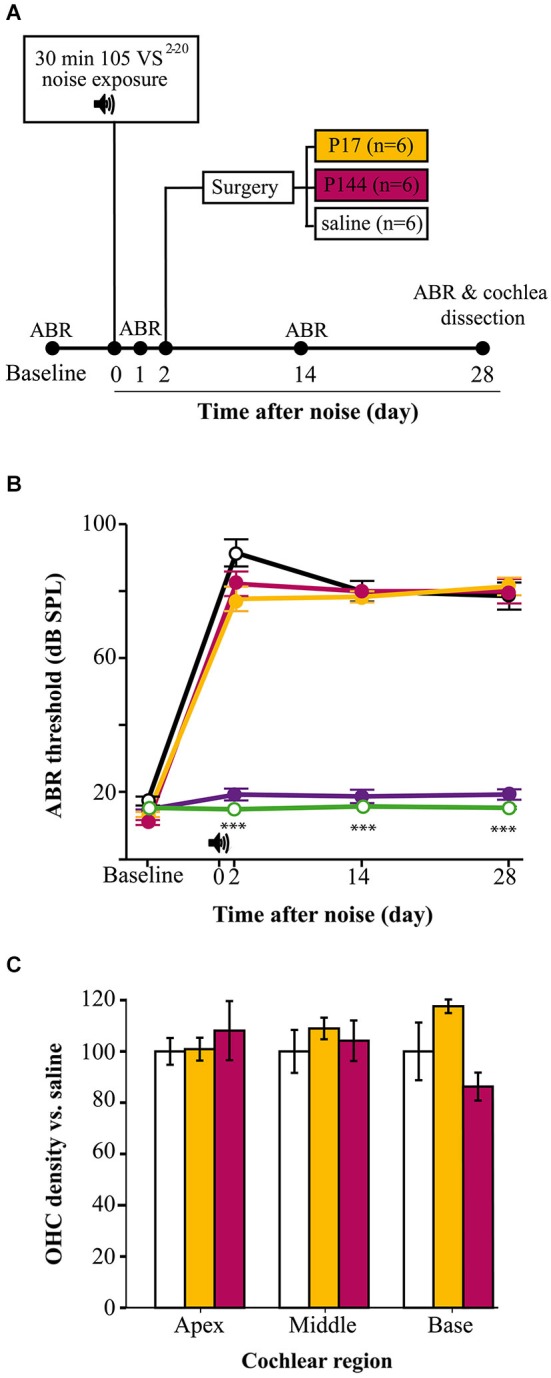
**TGF-β1** **inhibitors in NIHL treatment. (A)** Chronogram of the experiment. Mice were exposed for 30 min to VS noise at 105 dB SPL and after 2 days they were operated on to deliver P17, P144 or saline (*n* = 6 mice per group) to the inner ear through a bullostomy. ABR tests were performed before and 1, 14 and 28 days after noise exposure. **(B)** Evolution of threshold of click-evoked ABR in mice exposed to 105 VS^2–20^ noise for 30 min and treated locally with a single dose of TGF-β1 inhibitors P17 (orange line), P144 (pink) or saline (black), compared to non-exposed (green) and sham-operated (purple) mice . Noise induced notable TS in the first day after damage without noticeable recovery, compared to non-exposed controls (***p ≤ 0.001). No significant differences were observed between mice treated with TGF-β1 inhibitors and those treated with saline. No statistically significant increase in click-evoked ABR threshold was observed in sham-operated mice. **(C)** Outer hair cell density (mean ± SEM, expressed as %) in mice treated with TGF-β1 inhibitors compared to saline expressed as %) in mice treated with TGF-β1 inhibitors compared to saline treated mice, in the three standard regions (apex, 5–25; middle, 30–55; base, 60–80) at the end of the experiment. No significant differences were observed between mice treated with P17 (orange bars) or P144 (pink) and those treated with saline (white) after exposure.

NIHL mouse models and the cochleogram procedures were validated in a preclinical assay with TGF-β1 inhibitors (Figure 7A). Mice were exposed to 105 VS^2–20^ noise and after 2 days they were operated on for local delivery of a single dose of TGF-β1 inhibitor (P17, P144) or saline. Analysis of thresholds of click-evoked and tone-evoked ABR showed notable permanent TS 1 day after noise exposure and then a minimal recovery regardless of the treatment. No statistically significant differences were found in ABR thresholds in response to click or tone burst stimuli among mice treated with P17, P144 or saline at any of the times evaluated (1, 14 and 28 days after noise exposure) (Figure 7B).

At the end of the study, cochleae were collected to perform the cochleogram as described. As mentioned before, mice exposed to 105 VS^2–20^ noise showed a statistically significant decrease in HC number and density, both for IHCs and OHCs (data not shown). However, no significant differences were observed in cochleogram data between mice treated with TGF-β1 inhibitors and those with saline (Figure 7C).

## Discussion

NIHL is an important medical concern and reliable animal models are key to understanding its pathophysiology and developing new therapeutic strategies. In humans and mice, noise exposure induces temporary or permanent hearing loss depending on the noise intensity and the individual susceptibility (reviewed in Davis et al., 2003). Other physical parameters of noise as frequency spectra, duration and intermittency also contribute to injury, but they are less frequently examined in NIHL studies (Mahendra Prashanth and Venugopalachar, 2011) or animal models.

In this work we demonstrate that by modulating noise characteristics, the hearing loss outcome in noise-exposed mice can vary largely. Thus, by modifying the physical properties of the sound, we have generated and characterized a range of experimental NIHL mouse models with the aim of obtaining experimental tools for studying human NIHL and for testing potential therapeutic strategies.

During the first 2 weeks after noise exposure, mice can show temporary and reversible increased threshold (temporal TS), but one month after injury, the auditory threshold elevation is considered permanent (permanent TS) (Hamernik and Qiu, 2000; Harding et al., 2002; Wang et al., 2002; Chen et al., 2003). Here we show that 30 min of exposure to VS noise of intensities of either 105 or 120 dB SPL could induce a notable increase in thresholds of click-evoked and tone-evoked ABR; the magnitudes of temporal and permanent TSs are proportional to the intensity as reported (Ohlemiller et al., 2000, 2011; Park et al., 2013). Interestingly, small changes in the noise frequency spectrum also induced different ABR profiles. Two days after exposure to 105 VS^9–13^ there was an acute TS greater than that observed after exposure to 105 VS^2–20^ noise, particularly at high frequencies.

It is known that exposure to noise of a certain frequency does not induce a higher TS at that frequency, but rather at 0.5 to 2 octaves above that frequency, depending on the species (Ou et al., 2000a). Therefore, to assess functional impairment after noise exposure, it is necessary to measure thresholds of tone-evoked ABR over a range of frequencies by stimulation with multiple tone bursts. In this work we performed a large audiogram from 8 to 40 kHz. A notch was evident in the ABR audiogram from 105 VS^9–13^ noise-exposed mice with higher thresholds for pure tones at 8 and 10 kHz. Similar frequency-related variations in hearing thresholds have been reported in human studies. Exposures to broadband noise induce maximum TS at frequencies between 3 and 6 kHz but, if the noise is a pure tone, the higher the frequency, the greater the resulting TS (Mahendra Prashanth and Venugopalachar, 2011). In addition to level and frequency composition, other noise characteristics could influence the hearing outcome. Thus, swept-sine “intermittent” noises produce greater cochlear damage than “continuous” ones, including white, violet and broadband noises, possibly because they expose HCs to higher activity and stress.

Concomitantly with noise-induced TS, we observed a decrease in ABR peak latencies, which could be interpreted as acceleration in neural transmission along the auditory pathway. This observation is consistent with many studies both in human and animal models (Strelcyk et al., 2009; Scheidt et al., 2010; Henry et al., 2011), and diverse theories have been proposed, including recruitment phenomenon and hyperexcitability of central auditory fibers. Cochlear recruitment occurs when damaged HC call up those close to them to cover their activity, creating a paradoxical situation of hyperexcitability in the central auditory fibers, in which there is an abnormal increase in the response to sound (Moore, 2004; Sherlock and Formby, 2005). It is has been proposed that this is a mechanism for balancing neuronal activity after cochlear damage (Syka, 2002; Cai et al., 2009; Rybalko et al., 2011). However, the consequences of noise damage on ABR latencies are still unclear and other authors have observed increased values after exposure (Chen et al., 2002; Gourévitch et al., 2009). Wave amplitudes also decreased after noise-exposure, a pattern that was maintained throughout the study and which has been related to loss of afferent nerve terminals (Popelar et al., 2008; Kujawa and Liberman, 2009; Henry et al., 2011).

DPOAE is a method for early detection of NIHL in patients as their alteration may reflect subclinical cochlear changes that do not show up yet in the tone threshold audiometry (Attias et al., 2001; Martin et al., 2006; Fetoni et al., 2009). In parallel with changes in ABR parameters, we observed an increase in DPOAE thresholds in the three noise-exposed mice groups, without significant differences among them. DPOAE TS correlated with that observed in the ABR and also with the extent of OHC loss detected in the cochleogram, as described previously by other authors (Vázquez et al., 2004; Jamesdaniel et al., 2011; Park et al., 2013).

The CBA mouse strain has been reported to be more resistant to the harmful effects of noise than the C57 strain (Ohlemiller et al., 2011), a fact that was confirmed in our work by comparing two noise types, V and VS, at two intensities. C57 mice exposed to noise often showed an extreme response and became irreversibly profoundly deaf. Therefore CBA mice were used for further comparison of the lesions on different features caused by VS noises. CBA mice showed a characteristic noise-induced pattern of injury which includes changes in the spiral ligament, the limbus, the stria vascularis and the HC (Ou et al., 2000a; Wang et al., 2002; Ohlemiller et al., 2011; Park et al., 2013). In this study, in conjunction with the functional alterations observed, CBA mice exposed to VS noise showed cellular lesions in fibrocytes and HC. To fully characterize the effects of noise on HCs it is necessary to examine the entire OC from apex to base. However, surgical dissection of the organ of Corti is difficult, especially in the basal tip region or “hook”, which is usually lost or artificially damaged. We estimated that this portion represents around 20% of the total length of the OC, therefore the cochleogram includes the 80% of the cochlea that maintained cellular integrity. Based on the frequency location map of the mouse cochlea by Ou et al. (2000b), this portion results in a loss of information on the 50–80 kHz frequencies. As mentioned before, noise exposure induces higher TS in a range of frequencies 0.5 to 2 octaves around the noise frequency. Therefore this hook region should not be much affected by our VS. To further study HC loss resulting from different VS noise exposures, stereological methods were applied to develop a cytocochleogram, a topographical map of the cochlea which associates frequencies to defined HC positions along the length of the BM, offering key information on the function to structure relationship, under both physiological and pathological conditions (Viberg and Canlon, 2004). Due to intraspecies BM length variation, it is preferable to construct percentage, rather than absolute length (in mm) cochleograms. When length values are expressed as percentages, this results in a linear topographic map that can be safely used to correlate the location of the HC (or a lesion thereof) to sound frequency by using appropriate frequency-place equations (Viberg and Canlon, 2004). For this study with CBA mice, we chose to follow the Müller equation (Müller et al., 2005) and to plot the cytocochleogram dividing the length of the BM in 5% equidistant sectors from the apex to the base of the cochlea.

Cell counting procedures most often consist of direct counts of HC cell bodies or nuclei (or their absence from identified spots) at fixed length intervals from the apex, which may then be transformed into percentage distance from the cochlear apex (Nordmann et al., 2000; Minami et al., 2007; Harding and Bohne, 2009; Choudhury et al., 2011). We opted instead for estimating HC densities at regular 5% length intervals from the apex, an efficient and consistent novel procedure, which relied on applying a stereological method (Boyce et al., 2010) on phalloidin-stained stereocilia bundles as counting units. Single bundles are unambiguously identified on flat mounts of the cochlea, and their disappearance marks an irreversibly degenerating or already absent HC, since hair bundle loss is not recoverable in mammalian cochlear HC (Jia et al., 2009).

Using this cytocochleogram, we evidenced different patterns of HC loss depending on the level and frequency range of the VS noise, as well as a correlation with functional data. As reported for other sound stimuli in mice, VS noise induced a greater loss of OHC than IHC (Wang et al., 2002; Ohlemiller and Gagnon, 2007; Ohlemiller, 2008; Park et al., 2013). After exposure to noise with a particular range of frequencies, HC loss fundamentally occurs within the cochlear region that codifies this signal, but also outside this zone (Harding and Bohne, 2009). Consequently, we observed a notable HC loss, especially in OHC, in sectors 5–55% after exposure to 105 VS^2–20^ and a focal lesion in sectors 25–30% when 105 VS^9–13^ was used, but also we detected cell loss in the basal cochlear region. This is because the acoustic energy delivered by a noise is maximal in its frequency range and declines above and below it. As expected, after exposure to 120 VS^2–20^ noise the cochleogram showed generalized cochlear lesions with a marked decrease in HC density, with combined lesions in OHC and IHC and with large areas where no HC were detected.

The NIHL paradigm and cochleogram procedure shown here constitute valuable tools in preclinical assays of new molecules with potential therapeutic effects. Inhibitors are key regulators of the inflammatory and immune response in several tissues including the inner ear. Upregulation of TGF-β1 has been confirmed in some animal models with otic damage, including ototoxicity and antigen injection, chronic otitis media (Satoh et al., 2006; Wissel et al., 2006; Ghaheri et al., 2007) and NIHL (Murillo-Cuesta et al., submitted). In this context, the use of TGF-β1 inhibitors could be useful in ameliorating pathological changes in the cochlea after noise insult. Systemic treatment with P17 and P144 before noise exposure as well after damage has been shown to improve the TSs and the evolution of thresholds of click-evoked and tone-evoked ABR, compared to saline treated mice (Murillo-Cuesta et al., submitted). In order to deliver molecules to the inner ear with accuracy and to avoid adverse effects, new localized approaches to the cochlea are now being tested (Rivera et al., 2012). In this work we showed the functional and morphological results of a preclinical assay with TGF-β1 inhibitors applied to the middle ear using a gelatin sponge, after noise exposure. The potential therapeutic effect was evaluated with ABR and a cochleogram, as described. Mice exposed to noise showed a similar pattern of damage, both functional and morphological, with no significant differences compared to saline treated mice. Therefore, our results suggest that a single dose of these peptides is not enough to prevent noise damage.

In summary, we show that CBA mice exposed for 30 min to noise enriched in frequencies up to 20 kHz and presented in a swept-sine mode suffered a TS of around 50 dB. Depending on the intensity and frequency range, this noise induced specific functional and morphological changes, with a tonotopic correlation between the noise-frequency input and the injury output measured by means of functional data and cell density counting. The methodology described herein, combining standardized NIHL mouse models, friendly local delivery systems, and precise evaluation techniques such ABR and cochleogram, should be useful in further understanding NIHL and for evaluating novel therapeutic strategies.

## Authors and contributors

LS: acquisition, analysis, and interpretation of cytocochleogram data. SM-C: acquisition, analysis, and interpretation of functional data; drafting and revision of the manuscript. PC: substantial contribution to the conception and design of the noises and exposition chamber. RC: substantial contribution to the conception and design of the noises and exposition chamber. JC: acquisition, analysis, and interpretation of morphological data. TR: design of the work; revision of the manuscript. IVN: design of the work; analysis, and interpretation of data; drafting and revision of the manuscript. CA: design of the work, analysis and interpretation of data; critical revision of the manuscript.

## Conflict of interest statement

The authors declare that the research was conducted in the absence of any commercial or financial relationships that could be construed as a potential conflict of interest.

## Abbreviations

ABR: auditory brainstem responses
BM: basilar membrane
DPOAE: distortion product otoacoustic emissions
IHC: inner hair cell
NIHL: noise-induced hearing loss
OC: organ of Corti
OHC: outer hair cell
TS: threshold shift
V: violet noise
VS: violet swept-sine noise.

## References

[B1] Attias J. Horovitz G. El-Hatib N. Nageris B. (2001). Detection and clinical diagnosis of noise-induced hearing loss by otoacoustic emissions. Noise Health 3, 19–31. 12678938

[B2] Bohne B. A. Harding G. W. Lee S. C. (2007). Death pathways in noise-damaged outer hair cells. Hear. Res. 223, 61–70. 10.1016/j.heares.2006.10.004 17141990

[B3] Boyce R. W. Dorph-Petersen K. A. Lyck L. Gundersen H. J. (2010). Design-based stereology: introduction to basic concepts and practical approaches for estimation of cell number. Toxicol. Pathol. 38, 1011–1025. 10.1177/0192623310385140 21030683

[B4] Cai S. Ma W. L. Young E. D. (2009). Encoding intensity in ventral cochlear nucleus following acoustic trauma: implications for loudness recruitment. J. Assoc. Res. Otolaryngol. 10, 5–22. 10.1007/s10162-008-0142-y 18855070 PMC2644394

[B5] Cediel R. Riquelme R. Contreras J. Díaz A. Varela-Nieto I. (2006). Sensorineural hearing loss in insulin-like growth factor I-null mice: a new model of human deafness. Eur. J. Neurosci. 23, 587–590. 10.1111/j.1460-9568.2005.04584.x 16420467

[B6] Chen T. J. Chen S. S. Wang D. C. Hsieh Y. L. (2002). Increased vulnerability of auditory system to noise exposure in mdx mice. Laryngoscope 112, 520–525. 10.1097/00005537-200203000-00021 12148865

[B7] Chen G. D. Fechter L. D. (2003). The relationship between noise-induced hearing loss and hair cell loss in rats. Hear. Res. 177, 81–90. 10.1016/s0378-5955(02)00802-x 12618320

[B8] Chen Y. S. Liu T. C. Cheng C. H. Yeh T. H. Lee S. Y. Hsu C. J. (2003). Changes of hair cell stereocilia and threshold shift after acoustic trauma in guinea pigs: comparison between inner and outer hair cells. ORL J. Otorhinolaryngol. Relat. Spec. 65, 266–274. 10.1159/000075224 14730182

[B9] Choudhury B. Adunka O. F. Demason C. E. Ahmad F. I. Buchman C. A. Fitzpatrick D. C. (2011). Detection of intracochlear damage with cochlear implantation in a gerbil model of hearing loss. Otol. Neurotol. 32, 1370–1378. 10.1097/MAO.0b013e31822f09f2 21921858 PMC3338854

[B10] Cobo P. Murillo-Cuesta S. Cediel R. Moreno A. Lorenzo-García P. Varela-Nieto I. (2009). Design of a reverberant chamber for noise exposure experiments with small animals. Appl. Acoust. 70, 1034–1040 10.1016/j.apacoust.2009.03.005

[B11] Davis R. R. Kozel P. Erway L. C. (2003). Genetic influences in individual susceptibility to noise: a review. Noise Health 5, 19–28. 14558889

[B12] Ezquerro I. J. Lasarte J. J. Dotor J. Castilla-Cortázar I. Bustos M. Peñuelas I.. (2003). A synthetic peptide from transforming growth factor beta type III receptor inhibits liver fibrogenesis in rats with carbon tetrachloride liver injury. Cytokine 22, 12–20. 10.1016/s1043-4666(03)00101-7 12946101

[B13] Fetoni A. R. Garzaro M. Ralli M. Landolfo V. Sensini M. Pecorari G.. (2009). The monitoring role of otoacoustic emissions and oxidative stress markers in the protective effects of antioxidant administration in noise-exposed subjects: a pilot study. Med. Sci. Monit. 15, PR1–PR8. 19865065

[B14] Ghaheri B. A. Kempton J. B. Pillers D. A. Trune D. R. (2007). Cochlear cytokine gene expression in murine chronic otitis media. Otolaryngol. Head Neck Surg. 137, 332–337. 10.1016/j.otohns.2007.03.020 17666266

[B15] Gourévitch B. Doisy T. Avillac M. Edeline J. M. (2009). Follow-up of latency and threshold shifts of auditory brainstem responses after single and interrupted acoustic trauma in guinea pig. Brain Res. 1304, 66–79. 10.1016/j.brainres.2009.09.041 19766602

[B16] Gratton M. A. Eleftheriadou A. Garcia J. Verduzco E. Martin G. K. Lonsbury-Martin B. L.. (2011). Noise-induced changes in gene expression in the cochleae of mice differing in their susceptibility to noise damage. Hear. Res. 277, 211–226. 10.1016/j.heares.2010.12.014 21187137 PMC3098916

[B17] Greenwood D. D. (1996). Comparing octaves, frequency ranges and cochlear-map curvature across species. Hear. Res. 94, 157–162. 10.1016/0378-5955(95)00229-4 8789821

[B18] Hamernik R. P. Qiu W. (2000). Correlations among evoked potential thresholds, distortion product otoacoustic emissions and hair cell loss following various noise exposures in the chinchilla. Hear. Res. 150, 245–257. 10.1016/s0378-5955(00)00204-5 11077207

[B19] Harding G. W. Bohne B. A. (2009). Relation of focal hair-cell lesions to noise-exposure parameters from a 4- or a 0.5-kHz octave band of noise. Hear. Res. 254, 54–63. 10.1016/j.heares.2009.04.011 19393307

[B20] Harding G. W. Bohne B. A. Ahmad M. (2002). DPOAE level shifts and ABR threshold shifts compared to detailed analysis of histopathological damage from noise. Hear. Res. 174, 158–171. 10.1016/s0378-5955(02)00653-6 12433407

[B21] Henry K. S. Kale S. Scheidt R. E. Heinz M. G. (2011). Auditory brainstem responses predict auditory nerve fiber thresholds and frequency selectivity in hearing impaired chinchillas. Hear. Res. 280, 236–244. 10.1016/j.heares.2011.06.002 21699970 PMC3179834

[B22] Howard C. V. Reed M. G. (2005). Unbiased Stereology. Three Dimensional Measurement in Microscopy. 2nd Edn. New York: Garland Science/BIOS Scientific Publishers.

[B23] Hu B. H. Henderson D. Nicotera T. M. (2006). Extremely rapid induction of outer hair cell apoptosis in the chinchilla cochlea following exposure to impulse noise. Hear. Res. 211, 16–25. 10.1016/j.heares.2005.08.006 16219436

[B24] Jamesdaniel S. Hu B. Kermany M. H. Jiang H. Ding D. Coling D.. (2011). Noise induced changes in the expression of p38/MAPK signaling proteins in the sensory epithelium of the inner ear. J. Proteomics 75, 410–424. 10.1016/j.jprot.2011.08.007 21871588 PMC3225708

[B25] Jia S. Yang S. Guo W. He D. Z. (2009). Fate of mammalian cochlear hair cells and stereocilia after loss of the stereocilia. J. Neurosci. 29, 15277–15285. 10.1523/JNEUROSCI.3231-09.2009 19955380 PMC2795320

[B26] Kirchner D. B. Evenson E. Dobie R. A. Rabinowitz P. Crawford J. Kopke R.. (2012). Occupational noise-induced hearing loss: ACOEM task force on occupational hearing loss. J. Occup. Environ. Med. 54, 106–108. 10.1097/JOM.0b013e318242677d 22183164

[B27] Konings A. Van Laer L. Van Camp G. (2009). Genetic studies on noise-induced hearing loss: a review. Ear Hear. 30, 151–159. 10.1097/AUD.0b013e3181987080 19194285

[B28] Kujawa S. G. Liberman M. C. (2009). Adding insult to injury: cochlear nerve degeneration after “temporary” noise-induced hearing loss. J. Neurosci. 29, 14077–14085. 10.1523/JNEUROSCI.2845-09.2009 19906956 PMC2812055

[B29] Le Prell C. G. (2012). Noise-induced hearing loss: from animal models to human trials. Adv. Exp. Med. Biol. 730, 191–195. 10.1007/978-1-4419-7311-5_43 22278480

[B30] Mahendra Prashanth K. V. Venugopalachar S. (2011). The possible influence of noise frequency components on the health of exposed industrial workers–a review. Noise Health 13, 16–25. 10.4103/1463-1741.73996 21173483

[B31] Martin G. K. Stagner B. B. Lonsbury-Martin B. L. (2006). Assessment of cochlear function in mice: distortion-product otoacoustic emissions. Curr. Protoc. Neurosci. Chapter 8:Unit8.21C. 10.1002/0471142301.ns0821cs34 18428646

[B32] Minami S. B. Yamashita D. Ogawa K. Schacht J. Miller J. M. (2007). Creatine and tempol attenuate noise-induced hearing loss. Brain Res. 1148, 83–89. 10.1016/j.brainres.2007.02.021 17359945 PMC2680083

[B33] Moore B. C. (2004). Testing the concept of softness imperception: loudness near threshold for hearing-impaired ears. J. Acoust. Soc. Am. 115, 3103–3111. 10.1121/1.1738839 15237835

[B34] Müller M. Smolders J. W. (2005). Shift in the cochlear place-frequency map after noise damage in the mouse. Neuroreport 16, 1183–1187. 10.1097/00001756-200508010-00010 16012345

[B35] Müller M. von Hünerbein K. Hoidis S. Smolders J. W. (2005). A physiological place-frequency map of the cochlea in the CBA/J mouse. Hear. Res. 202, 63–73. 10.1016/j.heares.2004.08.011 15811700

[B36] Murillo-Cuesta S. Camarero G. González-Rodríguez A. De La Rosa L. R. Burks D. J. Avendano C.. (2012). Insulin receptor substrate 2 (IRS2)-deficient mice show sensorineural hearing loss that is delayed by concomitant protein tyrosine phosphatase 1B (PTP1B) loss of function. Mol. Med. 18, 260–269. 10.1016/s1096-6374(12)60045-8 22160220 PMC3324951

[B37] Murillo-Cuesta S. García-Alcántara F. Vacas E. Sistiaga J. A. Camarero G. Varela-Nieto I.. (2009). Direct drug application to the round window: a comparative study of ototoxicity in rats. Otolaryngol. Head Neck Surg. 141, 584–590. 10.1016/j.otohns.2009.07.014 19861195

[B39] Nordmann A. S. Bohne B. A. Harding G. W. (2000). Histopathological differences between temporary and permanent threshold shift. Hear. Res. 139, 13–30. 10.1016/s0378-5955(99)00163-x 10601709

[B40] Ohlemiller K. K. (2006). Contributions of mouse models to understanding of age- and noise-related hearing loss. Brain Res. 1091, 89–102. 10.1016/j.brainres.2006.03.017 16631134

[B41] Ohlemiller K. K. (2008). Recent findings and emerging questions in cochlear noise injury. Hear. Res. 245, 5–17. 10.1016/j.heares.2008.08.007 18790034 PMC2610263

[B42] Ohlemiller K. K. Gagnon P. M. (2007). Genetic dependence of cochlear cells and structures injured by noise. Hear. Res. 224, 34–50. 10.1016/j.heares.2006.11.005 17175124 PMC1809471

[B43] Ohlemiller K. K. Rybak Rice M. E. Rellinger E. A. Ortmann A. J. (2011). Divergence of noise vulnerability in cochleae of young CBA/J and CBA/CaJ mice. Hear. Res. 272, 13–20. 10.1016/j.heares.2010.11.006 21108998 PMC3465684

[B44] Ohlemiller K. K. Wright J. S. Heidbreder A. F. (2000). Vulnerability to noise-induced hearing loss in ‘middle-aged’ and young adult mice: a dose-response approach in CBA, C57BL and BALB inbred strains. Hear. Res. 149, 239–247. 10.1016/s0378-5955(00)00191-x 11033262

[B45] Ou H. C. Bohne B. A. Harding G. W. (2000a). Noise damage in the C57BL/CBA mouse cochlea. Hear. Res. 145, 111–122. 10.1016/s0378-5955(00)00081-2 10867283

[B46] Ou H. C. Harding G. W. Bohne B. A. (2000b). An anatomically based frequency-place map for the mouse cochlea. Hear. Res. 145, 123–129. 10.1016/s0378-5955(00)00082-4 10867284

[B47] Park S. N. Back S. A. Park K. H. Seo J. H. Noh H. I. Akil O.. (2013). Comparison of functional and morphologic characteristics of mice models of noise-induced hearing loss. Auris Nasus Larynx 40, 11–17. 10.1016/j.anl.2011.11.008 22364846

[B48] Popelar J. Grecova J. Rybalko N. Syka J. (2008). Comparison of noise-induced changes of auditory brainstem and middle latency response amplitudes in rats. Hear. Res. 245, 82–91. 10.1016/j.heares.2008.09.002 18812219

[B49] Riquelme R. Cediel R. Contreras J. la Rosa Lourdes R. D. Murillo-Cuesta S. Hernandez-Sanchez C.. (2010). A comparative study of age-related hearing loss in wild type and insulin-like growth factor I deficient mice. Front. Neuroanat. 4:27. 10.3389/fnana.2010.00027 20661454 PMC2907134

[B50] Rivera T. Sanz L. Camarero G. Varela-Nieto I. (2012). Drug delivery to the inner ear: strategies and their therapeutic implications for sensorineural hearing loss. Curr. Drug Deliv. 9, 231–242. 10.2174/156720112800389098 22283653

[B51] Rybalko N. Bureš Z. Burianová J. Popelář J. Grécová J. Syka J. (2011). Noise exposure during early development influences the acoustic startle reflex in adult rats. Physiol. Behav. 102, 453–458. 10.1016/j.physbeh.2010.12.010 21192960

[B52] Satoh H. Billings P. Firestein G. S. Harris J. P. Keithley E. M. (2006). Transforming growth factor beta expression during an inner ear immune response. Ann. Otol. Rhinol. Laryngol. 115, 81–88. 10.1177/000348940611500112 16466104

[B53] Scheidt R. E. Kale S. Heinz M. G. (2010). Noise-induced hearing loss alters the temporal dynamics of auditory-nerve responses. Hear. Res. 269, 23–33. 10.1016/j.heares.2010.07.009 20696230 PMC2934744

[B54] Sherlock L. P. Formby C. (2005). Estimates of loudness, loudness discomfort and the auditory dynamic range: normative estimates, comparison of procedures and test-retest reliability. J. Am. Acad. Audiol. 16, 85–100. 10.3766/jaaa.16.2.4 15807048

[B55] Sliwinska-Kowalska M. Davis A. (2012). Noise-induced hearing loss. Noise Health 14, 274–280. 10.4103/1463-1741.104893 23257577

[B56] Strelcyk O. Christoforidis D. Dau T. (2009). Relation between derived-band auditory brainstem response latencies and behavioral frequency selectivity. J. Acoust. Soc. Am. 126, 1878–1888. 10.1121/1.3203310 19813802

[B57] Syka J. (2002). Plastic changes in the central auditory system after hearing loss, restoration of function and during learning. Physiol. Rev. 82, 601–636. 10.1152/physrev.00002.2002 12087130

[B58] Vázquez A. E. Jimenez A. M. Martin G. K. Luebke A. E. Lonsbury-Martin B. L. (2004). Evaluating cochlear function and the effects of noise exposure in the B6.CAST+Ahl mouse with distortion product otoacoustic emissions. Hear. Res. 194, 87–96. 10.1016/s0378-5955(04)00130-3 15276680

[B59] Viberg A. Canlon B. (2004). The guide to plotting a cochleogram. Hear. Res. 197, 1–10. 10.1016/j.heares.2004.04.016 15504598

[B60] Wang Y. Hirose K. Liberman M. C. (2002). Dynamics of noise-induced cellular injury and repair in the mouse cochlea. J. Assoc. Res. Otolaryngol. 3, 248–268. 10.1007/s101620020028 12382101 PMC3202415

[B61] Wissel K. Wefstaedt P. Miller J. M. Lenarz T. Stöver T. (2006). Differential brain-derived neurotrophic factor and transforming growth factor-beta expression in the rat cochlea following deafness. Neuroreport 17, 1297–1301. 10.1097/01.wnr.0000233088.92839.23 16951573

