# Corrigendum to: Swept-sine noise-induced damage as a hearing loss model for preclinical assays

**DOI:** 10.3389/fnagi.2015.00079

**Published:** 2015-05-12

**Authors:** Lorena Sanz, Silvia Murillo-Cuesta, Pedro Cobo, Rafael Cediel, Julio Contreras, Teresa Rivera, Isabel Varela-Nieto, Carlos Avendaño

**Affiliations:** ^1^Príncipe de Asturias University Hospital, University of Alcalá, Alcalá de HenaresMadrid, Spain; ^2^Centre for Biomedical Network Research on Rare Diseases (CIBERER), Institute of Health Carlos III (ISCIII)Madrid, Spain; ^3^Institute for Biomedical Research “Alberto Sols” (IIBM), Spanish National Research Council–Autonomous University of Madrid (CSIC-UAM)Madrid, Spain; ^4^Hospital La Paz Institute for Health Research (IdiPAZ)Madrid, Spain; ^5^Instituto de Tecnologías Físicas y de la Información (ITEFI), Spanish National Research Council (CSIC)Madrid, Spain; ^6^Veterinary Faculty, Universidad Complutense de MadridMadrid, Spain; ^7^Department of Anatomy, Histology and Neuroscience, Medical School, Autónoma University of MadridMadrid, Spain

**Keywords:** cytocochleogram, hair cells, hearing loss, transtympanic, TGF-β inhibition, violet noise

Unfortunately, one of the affiliations included in the online published article is wrong. The complete and correct author's affiliation list is given here.

## Conflict of interest statement

The authors declare that the research was conducted in the absence of any commercial or financial relationships that could be construed as a potential conflict of interest.

